# Global burden and cross-country inequalities in stroke and subtypes attributable to diet from 1990 to 2019

**DOI:** 10.1186/s12889-024-19337-5

**Published:** 2024-07-08

**Authors:** Xian Chen, Jia Zheng, Jianying Wang, Hongping Wang, Hui Shi, Hongwei Jiang, Pengfei Shan, Qiang Liu

**Affiliations:** 1 Department of Geriatric Medicine, The Second Affiliated Hospital of Zhejiang University School of Medicine, 88 Jiefang Road, Hangzhou, 310009 Zhejiang China; 2 Department of Rehabilitation Medicine, The Second Affiliated Hospital of Zhejiang University School of Medicine, Hangzhou, China; 3 Department of Endocrinology, The First Affiliated Hospital, and College of Clinical Medicine of Henan University of Science and Technology, Luoyang, China; 4 Department of Endocrinology and Metabolism, The Second Affiliated Hospital of Zhejiang University School of Medicine, Binjiang Institute of Zhejiang University, 88 Jiefang Road, Hangzhou, 310009 Zhejiang China

**Keywords:** Stroke, Mortality, Years lived with Disability (YLDs), Health inequality, Dietary risks, Global burden of disease (GBD)

## Abstract

**Data sources:**

The Global Burden of Diseases, Injuries, and Risk Factors study (GBD) 2019.

**Background:**

To describe burden, and to explore cross-country inequalities according to socio-demographic index (SDI) for stroke and subtypes attributable to diet.

**Methods:**

Death and years lived with disability (YLDs) data and corresponding estimated annual percentage changes (EAPCs) were estimated by year, age, gender, location and SDI. Pearson correlation analysis was performed to evaluate the connections between age-standardized rates (ASRs) of death, YLDs, their EAPCs and SDI. We used ARIMA model to predict the trend. Slope index of inequality (SII) and relative concentration index (RCI) were utilized to quantify the distributive inequalities in the burden of stroke.

**Results:**

A total of 1.74 million deaths (56.17% male) and 5.52 million YLDs (55.27% female) attributable to diet were included in the analysis in 2019.Between 1990 and 2019, the number of global stroke deaths and YLDs related to poor diet increased by 25.96% and 74.76% while ASRs for death and YLDs decreased by 42.29% and 11.34% respectively. The disease burden generally increased with age. The trends varied among stroke subtypes, with ischemic stroke (IS) being the primary cause of YLDs and intracerebral hemorrhage (ICH) being the leading cause of death. Mortality is inversely proportional to SDI (*R* = -0.45, *p* < 0.001). In terms of YLDs, countries with different SDIs exhibited no significant difference (*p* = 0.15), but the SII changed from 38.35 in 1990 to 45.18 in 2019 and the RCI showed 18.27 in 1990 and 24.98 in 2019 for stroke. The highest ASRs for death and YLDs appeared in Mongolia and Vanuatu while the lowest of them appeared in Israel and Belize, respectively. High sodium diets, high red meat consumption, and low fruit diets were the top three contributors to stroke YLDs in 2019.

**Discussion:**

The burden of diet-related stroke and subtypes varied significantly concerning year, age, gender, location and SDI. Countries with higher SDIs exhibited a disproportionately greater burden of stroke and its subtypes in terms of YLDs, and these disparities were found to intensify over time. To reduce disease burden, it is critical to enforce improved dietary practices, with a special emphasis on mortality drop in lower SDI countries and incidence decline in higher SDI countries.

## Introduction

Stroke is a significant cause of long-term disability and death around the world, with profound implications for human health [1]. Survivors often require assistance in daily activities due to physical and mental impairments, such as limb weakness, speech difficulties, fatigue, and cognitive challenges [2]. With the aging global population and increasing life expectancy, these statistics are expected to rise, imposing a significant economic burden on individuals, families, and communities [3]. Thus, the prevention of stroke through modifiable risk factors is vital for public health.

Dietary risks have received a lot of attention in recent years due to their significant impact on stroke risk. High sodium diets have been identified in multiple studies as a significant risk factor for stroke [4–7]. A modeling study revealed that adhering to the World Health Organization’s (WHO’s) recommendation for salt intake (5 g/day) could prevent 126 thousand premature stroke cases by 2050 in the United Kingdom [8]. The Salt Substitute and Stroke Study showed that salt substitution could reduce stroke risk by 14% and provide cost-effective and cost-saving policy options in China [9]. Furthermore, increased consumption of fruits and vegetables, especially apples, pears, and green leafy vegetables, has been associated with reduced stroke risk in a Swedish study [10].

While the focus has been on high sodium intake and fruit/vegetable consumption, other dietary patterns such as the Dietary Approaches to Stop Hypertension (DASH) diet and the Mediterranean diet also warrant consideration. The DASH diet, rich in fruits, vegetables, and low-fat dairy products, has been shown to lower blood pressure effectively [11]. Similarly, the Mediterranean diet, characterized by high intake of plant-based foods, fish, and healthy fats like olive oil, is associated with a reduced risk of cardiovascular diseases, including stroke [11]. Integrating these diets into public health strategies could provide additional avenues for stroke prevention.

However, research has not yet determined if specific dietary risks have similar impacts across different subtypes of strokes, such as ischemic stroke (IS), intracerebral hemorrhage (ICH) and subarachnoid hemorrhage (SAH). The influence of diet on stroke risk may vary globally, due to regional dietary habits and genetic factors. t Since stroke subtypes have distinct underlying mechanisms, tailored nutritional interventions may bring about specific benefits. Thus, a comprehensive evaluation of trends in mortality and years lived with disability (YLDs) for stroke and its subtypes attributable to diet is urgently needed.

Moreover, using the World Health Organization's (WHO) standard health equity analytic methods, we aim to assess potential sociodemographic development level-related inequalities in the burden of stroke and subtypes across countries, as well as their magnitude and trends over time.. This data is critical for developing cost-effective policies and tailored interventions for primary stroke prevention, particularly in high-risk populations.

## Methods

### Data source and definitions

Using the most recent epidemiological data and improved standardized methodologies, the Global Burden of Diseases, Injuries, and Risk Factors study (GBD) 2019 provided a thorough evaluation on the health loss for 369 diseases, injuries, and impairments and 87 risk factors across 204 countries and territories [12, 13]. Considering the similar trends of death and disability-adjusted life years (DALYs), our study used the following two epidemiological measures: death and YLDs, to describe the disease burden. YLDs, which are parts of DALYs, are estimated by multiplying stroke prevalence with the corresponding disability wights [14].Our study used the records included relevant information on stroke deaths and YLDs attributable to dietary risks, together with the related crude and age-standardized rates (ASRs) by factors such age, gender, SDI, and region.

In instances where it became necessary to contrast populations from disparate areas or those that present varying age patterns over time, ASR was derived through weighted averaging of age-specific rates. To signify age-standardized mortality rate (ASMR) and age-standardized YLDs rate (ASYR) trends spanning from 1990 to 2019, we computed the EAPC utilizing the formula

$$Y = \alpha + \beta X + \varepsilon$$

where Y = ln (ASR), X = calendar year, and ε = the error term.

$$EAPC = 100\times(exp(\beta)-1)$$

Based on linear regression models, we obtained a 95% confidence interval (CI) for our findings [15]. When both EAPC estimations and 95% CI lower limits were positive, the ASR trended upward. When both EAPC estimations and 95% CI upper limit were negative, the ASR decreased. The trend remained stable during the study period if neither of these conditions were met [5].

Dietary risks associated with stroke include 6 factors: low consumption of fruits (200–300 g/day), vegetables (290–430 g/day), whole grains (100–150 g/day), and fiber (19–28 g/day), and high intake of red meat (18–27 g/day) and sodium (1–5 g/day).

Since our study was based on publicly available data, there was no need for ethical approval or informed consent.

### Socio-demographic index

The Socio-demographic Index (SDI) was used in the study as a tool for assessing the socio-economic standing of various countries. Higher scores indicate more robust socioeconomic development [16–19]. The SDI is generated from country-level per capita income, average educational attainment, and total fertility rate, and has a possible range of values from 0 to 1. According on SDI, countries were divided into five quintiles for the GBD Study of 2019: high SDI (> 0.81), high-middle SDI (0.70–0.81), middle SDI (0.61–0.69), low-middle SDI (0.46–0.60), and low SDI (0.46) [16]. In order to ease the analysis, the study divided similarly situated nations and territories into 21 areas. Using Pearson's correlation analysis, the relationships between ASMR, ASDR, their EAPCs and SDIs were calculated.

### Method for forecasting stroke burden beyond 2019

The Auto-Regressive Integrated Moving Average (ARIMA) model is a popular technique used in epidemiology for forecasting time-series data [20]. In our study, we constructed an ARIMA model using the R programming language to predict the burden of stroke from 2020 to 2050.

### Cross-country inequality analysis

Slope index of inequality (SII) and relative concentration index (RCI), which are two standard metrics of absolute and relative gradient inequality, respectively, were applied to quantify the distributive inequality of the burden of stroke and subtypes across countries. Positive values suggest concentration among the advantaged, while negative values indicate concentration among the disadvantaged. SII is calculated by regressing national YLDs rates on an SDI-based relative position scale. Higher absolute values indicate greater inequality. RCI is obtained by integrating under the Lorenz concentration curve. Larger absolute RCI values indicate higher levels of inequality [21].

### Statistical analysis

The data in our study is presented as values with 95% uncertainty intervals (UI). Crude and age-standardized death and YLD rates are expressed as the number per 100,000 population. We examined the association between SDI and disease burden using Pearson correlation analysis and the curve fitting approach. We used R language (Version 4.2.1) and the Health Equity Assessment Toolkit (HEAT) from WHO for all statistical analysis and visualizations of results. A *p*-value of less than 0.05 was considered statistically significant.

## Results

### Global trend of total and gender-specific stroke burden by year and age

**Fig. 1:**
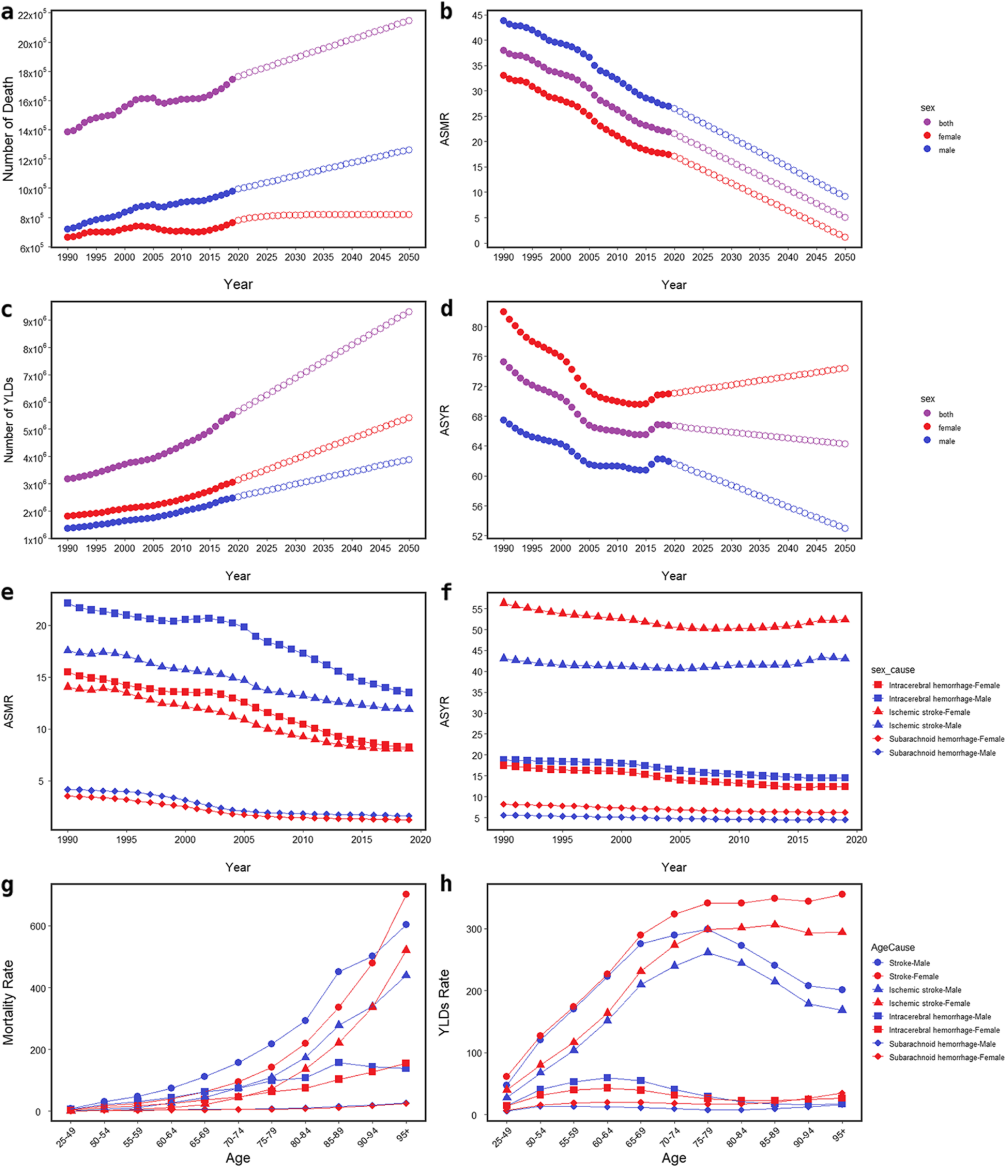
**a**–**d** Global burden of stroke attributable to diet from 1990 to 2050. **e** & **f** Global burden of subtypes attributable to diet from 1990 to 2019 by gender. **g** & **h** Global burden of stroke and subtypes attributable to diet by age in 2019. *YLDs *years lived with disability, *ASMR *Age-standardized mortality rate, *ASYR *Age-standardized YLDs rate

 In 2019, dietary risks were responsible for 1.74 (95% UI:1.24,2.36) million deaths and 5.52 (95% UI:3.53,7.98) million YLDs attributable to stroke, marking a 25.96% increase in deaths and 74.76% in YLDs since 1990. Despite these increases, the ASMR and ASYR of both genders had decreased by 42.29% and 11.34%, respectively. The ARIMA model forecasts a potential rise in stroke burden, predicting 2.15 million deaths and 9.30 million YLDs by 2050 without effective interventions (Fig. 1a, c). Additionally, according to the ARIMA model, the trend of ASYR in females will gradually increase and reach 74.39 per 100,000 population in 2050 (Fig. 1d).

Males accounted for a higher proportion of stroke deaths (56.17%), while females made up the majority of YLDs (55.27%) (Fig. 1a, c). The ASMR for both genders reached their lowest points in 2019 (males 26.95 [95% UI:18.75,36.81] vs females 17.46 [95% UI:12.09,24.39]) per 100,000 population. In 2014, the ASYR for both genders reached their lowest points (males 60.73 [95% UI:39.53,87.28] vs females 69.56 [95% UI:44.93,101.19]) per 100,000 population (Fig. 1b, d).

The ASMR changes of the three subtypes were consistent with those of total stroke. In 2019, ICH had the highest ASMR, followed by IS and SAH, with males experiencing more severe outcomes than females (Fig. 1e). The IS had the least reduction (EAPC of -1.85 [95% CI: -1.94, 1.76]), and SAH had the most significant decline (EAPC of -4.03 [95% CI: -4.39, -3.68]) (Fig. 3). On the other hand, IS had the highest ASYR, followed by ICH and SAH, with females experiencing more severe outcomes than males, except in cases of ICH (Fig. 1f). The IS also had the least decrease (EAPC of -0.16 [95% CI: -0.25, -0.07]), while ICH had a substantial drop (EAPC of -1.23 [95% CI: -1.31, -1.14]) (Fig. 3).

In 2019, the stroke mortality rate per 100,000 population demonstrated a positive correlation with age for both sexes, with a noticeable rise after the age of 80 years, and peaking at the 95 plus age group. Males consistently experienced a higher mortality rate compared to females within the same age group, until females surpassed males at the 95 plus age group with a mortality rate of 701.63 (95% UI: 448.80, 1025.09) deaths per 100,000 population. A similar trend was observed in the three subtypes. However, among males, the mortality rate of ICH reached its zenith within the 85–89 age group, whereas the mortality rate of SAH was consistently higher than that of females within the corresponding age groups (Fig. 1g). Concerning the YLDs rate of stroke per 100,000 population, it increased with age, reaching its peak at the 75–79 age group for males (298.98 [95% UI: 168.42, 482.58]) and the 95 plus age group for females (355.37 [95% UI: 204.16, 580.86]). Except for those under 80 affected by ICH, females consistently had a higher YLDs rate than males within the same age group, for both total stroke and subtypes. Specifically, for IS, females peaked at the 85–89 age group, while males peaked at the 75–79 age group. As for ICH, the YLDs rate for both genders peaked at the 60–64 age group. Regarding SAH, the YLDs rate for both genders was highest in the 95 plus age group (Fig. 1h).

### Stroke burden attributable to diet by socioeconomic status

**Fig. 2:**
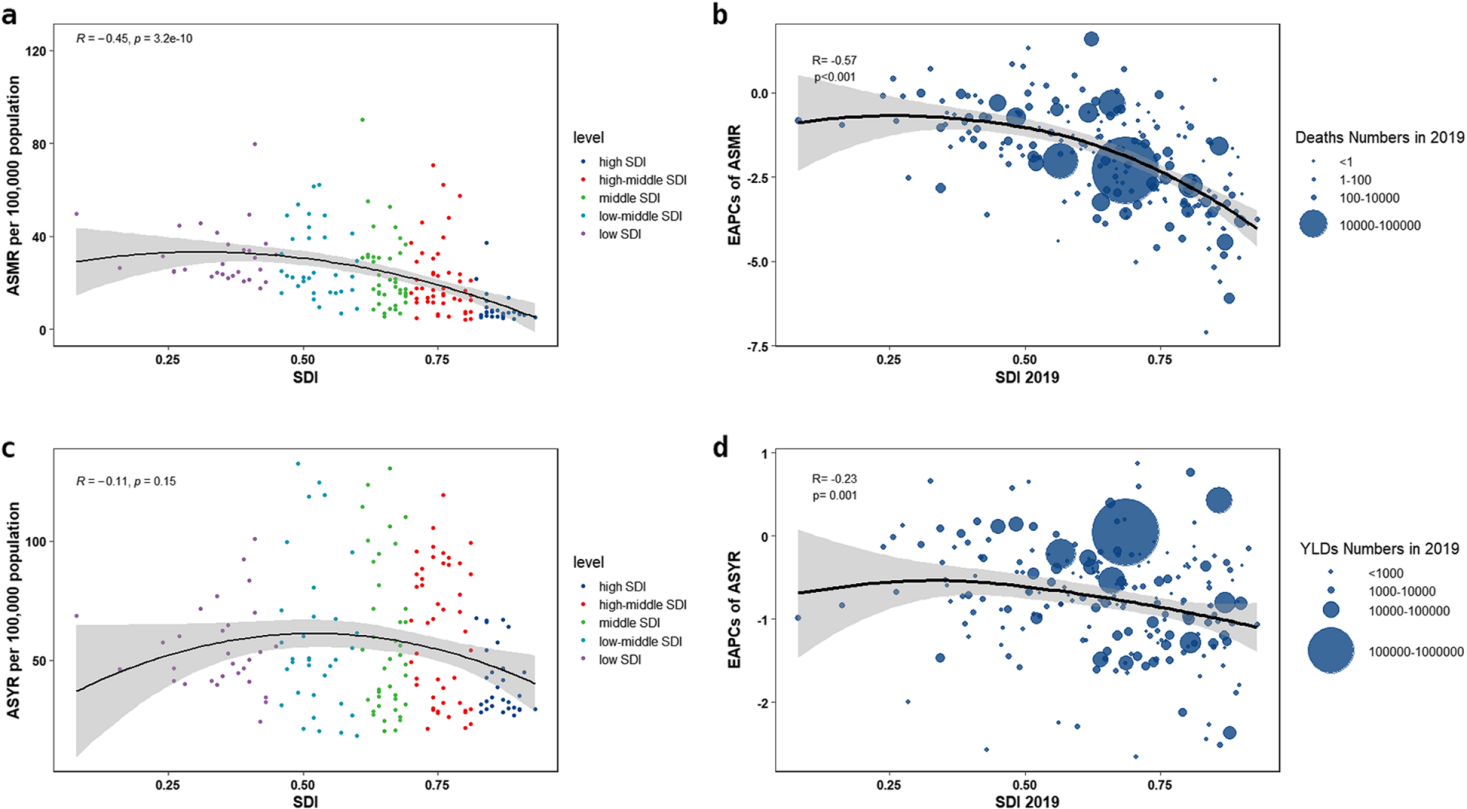
The correlations between ASMR (**a**), ASYR (**c**) and SDI of 204 countries and territories in 2019. The correlations between EAPCs of ASMR(**b**), ASYR(**d**)from 1990–2019 and SDI in 2019, the size of circle represents the numbers of stroke deaths or YLDs in 2019. *YLDs *years lived with disability, *ASMR *Age-standardized mortality rate, *ASYR *Age-standardized YLDs rate, *EAPC *estimated annual percentage change, *SDI *socio-demographic index

In 2019, the middle SDI quintile had the highest ASMR, four times higher than the high SDI quintile, which had the lowest ASMR (Table 1). A negative correlation between ASMR and SDI was observed across 204 countries and territories (*R* = -0.45, *p* < 0.001, Fig. 2a). In terms of ASYR, the middle SDI quintile also had the highest ASYR, which was around twice higher than the low SDI quintile with the lowest ASYR (Table 2). However, there were no significant correlation with SDI was found(*p* = 0.15, Fig. 2c).

**Table 1:** Age-standardized mortality rate (per 100,000 population) of stroke attributable to diet by gender, subtypes, SDI and GBD Super Region, 1990 and 2019

	**1990**												**2019**											
	**Stroke**			**ICH**			**IS**			**SAH**			**Stroke**			**ICH**			**IS**			**SAH**		
	**B**	**M**	**F**	**B**	**M**	**F**	**B**	**M**	**F**	**B**	**M**	**F**	**B**	**M**	**F**	**B**	**M**	**F**	**B**	**M**	**F**	**B**	**M**	**F**
**Global**	38.0	43.8	33.0	18.5	22.1	15.5	15.6	17.5	14.0	3.8	4.2	3.5	21.9	27.0	17.5	10.7	13.5	8.2	9.8	11.9	8.0	1.4	1.6	1.2
**SDI quintile**																								
High SDI	18.4	21.7	15.8	5.8	7.3	4.6	10.8	12.9	9.4	1.7	1.5	1.8	7.6	9.1	6.3	2.6	3.4	2.0	4.0	4.7	3.4	1.0	1.0	0.9
High-middle SDI	46.5	55.9	39.3	19.3	24.9	15.1	24.0	27.2	21.5	3.2	3.8	2.8	23.3	30.3	17.6	8.8	12.3	6.0	13.3	16.6	10.6	1.2	1.4	1.0
Middle SDI	50.4	56.3	44.8	28.0	31.4	24.8	15.0	17.2	13.2	7.3	7.7	6.9	28.9	36.6	22.1	15.2	19.5	11.2	12.2	15.1	9.6	1.6	1.9	1.3
Low-middle SDI	40.2	43.6	36.7	24.6	26.5	22.5	11.6	12.8	10.3	4.0	4.2	3.8	27.1	31.1	23.5	15.5	17.7	13.4	9.8	11.4	8.4	1.9	2.1	1.7
Low SDI	33.4	35.5	31.2	22.5	24.0	20.8	8.9	9.4	8.4	2.0	2.1	1.9	24.3	25.1	23.6	14.9	15.3	14.4	8.1	8.3	7.9	1.3	1.4	1.3
**High-income**																								
High-income Asia Pacific	26.8	32.8	22.4	9.7	12.0	7.7	14.5	18.4	11.8	2.7	2.4	2.9	7.5	9.8	5.5	2.7	3.7	1.7	3.6	4.8	2.7	1.2	1.3	1.1
Western Europe	17.9	21.1	15.7	4.8	5.9	3.9	11.9	14.1	10.5	1.2	1.1	1.3	6.9	7.9	6.1	2.1	2.7	1.7	4.0	4.5	3.6	0.7	0.7	0.7
Australasia	15.3	16.7	14.0	4.0	4.7	3.4	9.7	10.7	8.9	1.5	1.3	1.7	6.1	6.4	5.8	1.8	2.1	1.5	3.5	3.5	3.4	0.8	0.8	0.9
High-income North America	10.5	11.4	9.8	3.1	3.8	2.6	6.1	6.6	5.7	1.3	1.1	1.4	6.9	7.5	6.2	2.6	3.2	2.1	3.2	3.2	3.1	1.1	1.1	1.0
Southern Latin America	30.5	36.2	25.7	14.1	17.7	11.0	13.2	15.4	11.5	3.1	3.1	3.2	12.8	15.5	10.6	5.3	6.9	3.9	5.9	7.0	5.1	1.6	1.5	1.6
**Eastern Europe&Central Asia**																								
Central Europe	55.7	67.6	47.0	18.5	23.3	14.7	34.6	41.3	29.8	2.7	3.0	2.5	27.9	36.0	21.9	7.7	10.8	5.4	18.4	23.1	15.1	1.8	2.1	1.5
Eastern Europe	46.8	57.0	41.0	9.9	12.5	8.2	35.1	42.1	31.5	1.8	2.4	1.4	28.7	37.2	22.7	6.6	9.6	4.5	20.2	25.3	16.9	1.8	2.4	1.3
Central Asia	47.2	56.9	40.8	22.3	27.0	19.0	23.0	27.5	20.3	1.9	2.4	1.5	39.8	50.1	32.5	18.1	23.3	14.3	19.9	24.5	16.8	1.8	2.3	1.4
**Latin America&Caribbean**																								
Andean Latin America	17.9	19.3	16.5	9.0	10.2	7.8	6.4	6.9	5.9	2.5	2.2	2.8	8.5	9.3	7.9	3.5	4.0	3.0	3.6	3.9	3.3	1.5	1.3	1.6
Central Latin America	15.4	16.7	14.3	7.0	7.6	6.5	7.0	7.8	6.3	1.4	1.3	1.5	8.8	10.4	7.4	3.7	4.7	2.9	3.8	4.5	3.2	1.3	1.3	1.3
Tropical Latin America	37.0	43.3	31.4	15.9	18.8	13.2	18.3	22.1	15.1	2.8	2.5	3.1	15.3	18.7	12.6	5.8	7.3	4.5	7.7	9.8	6.0	1.8	1.6	2.1
Caribbean	21.1	21.8	20.5	10.5	11.3	9.7	9.1	9.1	9.0	1.6	1.4	1.8	14.6	15.7	13.6	6.9	7.8	6.1	6.4	6.8	6.1	1.2	1.1	1.4
**Southeast Asia, East Asia and Oceania**																								
Southeast Asia	56.6	63.9	50.1	35.9	40.1	32.0	17.2	20.0	14.8	3.5	3.8	3.2	39.8	47.9	32.7	22.4	27.3	18.0	15.5	18.4	13.0	1.9	2.2	1.6
East Asia	70.4	85.6	58.8	40.9	49.9	33.8	18.6	23.4	15.1	10.9	12.3	9.9	36.0	49.9	24.8	18.7	26.0	12.6	15.6	21.7	10.9	1.6	2.2	1.2
Oceania	40.8	49.8	31.9	28.9	36.8	21.0	8.1	9.9	6.4	3.8	3.0	4.5	35.3	41.2	29.3	25.0	30.5	19.4	7.5	8.4	6.6	2.8	2.3	3.3
**South Asia**																								
South Asia	31.4	33.4	29.3	18.8	19.8	17.8	9.6	10.6	8.5	3.0	3.0	3.0	19.7	21.6	17.8	11.1	12.0	10.2	6.9	7.8	6.0	1.7	1.8	1.6
**North Africa and Middle East**																								
North Africa and Middle East	19.2	19.4	19.0	7.0	7.2	6.8	11.2	11.2	11.0	1.1	1.0	1.2	12.8	12.7	12.9	2.9	3.0	2.8	9.5	9.3	9.6	0.4	0.4	0.4
**Sub-Saharan Africa**																								
Southern Sub-Saharan Africa	21.4	22.7	20.2	12.6	13.2	11.8	8.2	8.7	7.8	0.7	0.8	0.6	18.8	20.1	17.5	9.1	10.2	8.1	9.1	9.3	8.9	0.6	0.7	0.5
Eastern Sub-Saharan Africa	42.2	46.3	38.1	31.5	35.2	27.8	9.1	9.2	8.9	1.6	2.0	1.3	29.1	30.3	27.9	19.1	20.2	18.1	8.9	8.8	8.9	1.1	1.3	0.9
Central Sub-Saharan Africa	29.4	32.9	26.5	20.7	23.5	18.2	7.9	8.4	7.6	0.9	1.1	0.7	24.9	27.3	22.9	16.0	17.8	14.5	8.0	8.4	7.7	0.8	1.0	0.7
Western Sub-Saharan Africa	26.1	25.5	26.4	18.1	18.1	17.9	7.4	6.7	8.0	0.6	0.7	0.5	19.6	19.9	19.4	13.0	13.5	12.5	6.3	6.0	6.6	0.4	0.5	0.3

*T* total, *F* female, *M* male, *ICH* intracerebral hemorrhage, *IS* ischemic stroke, *SAH* subarachnoid hemorrhage

**Table 2:** Age-standardized YLDs rate (per 100,000 population) of stroke attributable to diet by gender, subtypes, SDI and GBD Super Region, 1990 and 2019

	**1990**												**2019**											
	**Stroke**			**ICH**			**IS**			**SAH**			**Stroke**			**ICH**			**IS**			**SAH**		
	**B**	**M**	**F**	**B**	**M**	**F**	**B**	**M**	**F**	**B**	**M**	**F**	**B**	**M**	**F**	**B**	**M**	**F**	**B**	**M**	**F**	**B**	**M**	**F**
**Global**	75.3	67.5	81.9	18.1	18.8	17.5	50.2	43.1	56.3	7.0	5.6	8.2	66.7	61.9	70.9	13.4	14.5	12.4	47.9	43.0	52.3	5.4	4.5	6.2
**SDI quintile**																								
High SDI	62.6	56.9	67.3	9.2	9.1	9.2	45.2	42.1	47.7	8.2	5.8	10.4	53.2	48.6	57.7	7.0	6.9	7.1	38.4	36.1	40.6	7.8	5.5	10.0
High-middle SDI	89.0	81.7	95.1	18.3	20.5	16.5	62.8	54.6	69.5	7.9	6.5	9.0	76.2	72.3	79.5	12.1	14.0	10.3	58.1	53.1	62.6	5.9	5.2	6.6
Middle SDI	87.8	77.9	96.9	25.2	26.2	24.3	55.3	45.4	64.4	7.3	6.3	8.2	80.6	75.0	85.5	17.2	19.5	15.1	58.3	50.9	64.9	5.1	4.6	5.5
Low-middle SDI	54.2	48.5	60.0	18.1	17.4	18.7	31.6	27.1	36.1	4.6	4.0	5.2	50.8	46.7	54.7	14.4	14.3	14.6	32.7	29.1	36.0	3.7	3.3	4.1
Low SDI	49.0	42.1	55.8	17.0	15.8	18.3	28.9	23.7	34.1	3.0	2.6	3.4	43.4	37.2	49.5	13.2	12.2	14.0	27.7	22.7	32.5	2.6	2.3	2.9
**High-income**																								
High-income Asia Pacific	87.8	84.7	90.0	17.1	17.4	16.6	56.3	57.5	55.3	14.5	9.8	18.2	66.8	63.3	69.7	10.7	11.7	9.8	40.6	40.9	40.1	15.5	10.7	19.8
Western Europe	41.2	39.1	43.1	5.5	5.5	5.6	31.0	29.6	32.3	4.7	4.0	5.3	30.9	28.3	33.3	3.9	3.8	4.0	22.8	20.8	24.7	4.1	3.7	4.6
Australasia	44.7	38.3	50.5	4.9	4.2	5.5	35.0	30.4	39.0	4.8	3.6	6.0	32.8	25.7	39.3	3.6	2.9	4.2	25.0	19.6	30.0	4.2	3.2	5.1
High-income North America	61.0	50.0	70.0	6.0	5.1	6.8	47.2	40.4	52.5	7.7	4.4	10.7	58.0	51.9	63.7	6.0	5.0	7.0	44.6	42.4	46.7	7.3	4.5	9.9
Southern Latin America	57.6	51.9	62.3	14.0	14.1	13.9	37.1	32.9	40.6	6.4	4.9	7.8	39.8	34.3	44.5	8.3	8.1	8.5	26.9	22.6	30.5	4.6	3.6	5.6
**Eastern Europe&Central Asia**																								
Central Europe	116.4	109.0	122.4	17.9	21.4	15.1	87.8	79.4	94.6	10.7	8.2	12.7	79.0	74.1	83.8	10.0	11.3	8.9	61.0	56.2	65.6	8.0	6.6	9.3
Eastern Europe	101.5	88.7	109.5	14.7	16.2	13.8	76.2	64.0	84.1	10.5	8.5	11.7	76.0	69.4	81.3	11.6	13.0	10.7	55.3	48.8	60.6	9.0	7.6	10.0
Central Asia	96.5	87.7	102.2	26.3	29.8	23.6	62.5	50.8	70.8	7.7	7.2	7.9	72.3	67.7	75.8	18.3	21.1	15.9	48.1	40.9	53.9	6.0	5.7	6.0
**Latin America&Caribbean**																								
Andean Latin America	31.6	26.9	36.2	8.3	7.4	9.2	18.8	16.2	21.4	4.5	3.3	5.6	24.7	21.8	27.4	5.4	4.9	5.8	15.8	14.1	17.3	3.5	2.8	4.3
Central Latin America	33.0	26.3	39.4	6.9	5.2	8.6	23.0	18.8	26.8	3.1	2.2	4.0	25.5	21.2	29.4	5.0	4.0	5.9	17.7	15.1	20.0	2.8	2.1	3.4
Tropical Latin America	55.8	51.4	59.8	14.9	12.5	17.0	35.8	35.8	35.8	5.1	3.1	7.0	37.7	34.0	41.1	9.2	7.3	10.9	24.5	24.4	24.7	4.0	2.4	5.4
Caribbean	31.0	26.2	35.5	6.6	5.6	7.6	21.9	19.0	24.6	2.5	1.7	3.3	25.9	22.1	29.4	5.1	4.4	5.8	18.7	16.3	20.9	2.1	1.4	2.7
**Southeast Asia, East Asia and Oceania**																								
Southeast Asia	127.6	124.9	129.9	45.9	50.8	41.6	71.9	65.2	77.6	9.8	8.8	10.7	105.2	104.1	106.0	33.0	37.6	28.9	64.8	59.7	69.2	7.4	6.8	7.9
East Asia	107.1	96.1	116.7	28.3	30.9	25.6	69.8	57.1	81.2	9.0	8.1	9.9	109.3	102.1	115.4	17.7	21.7	13.8	85.2	74.4	94.7	6.4	6.0	6.9
Oceania	102.3	94.5	110.1	41.1	44.3	37.7	51.0	42.9	59.2	10.2	7.3	13.1	96.5	87.5	105.4	38.3	40.5	35.7	49.1	40.2	58.0	9.1	6.8	11.6
**South Asia**																								
South Asia	37.5	32.9	42.5	13.2	11.3	15.3	20.6	18.4	23.1	3.6	3.2	4.1	35.3	32.0	38.7	11.6	10.2	13.1	20.6	18.9	22.3	3.1	2.8	3.4
**North Africa and Middle East**																								
North Africa and Middle East	47.8	39.6	56.1	7.9	7.0	8.9	37.6	31.0	44.3	2.2	1.6	2.9	44.0	37.3	51.1	5.5	5.1	5.9	36.9	31.0	43.2	1.6	1.3	2.0
**Sub-Saharan Africa**																								
Southern Sub-Saharan Africa	57.5	50.7	63.0	14.6	15.0	14.2	40.9	34.0	46.5	2.0	1.7	2.3	52.2	45.6	57.1	11.0	11.4	10.6	39.2	32.6	44.3	2.0	1.6	2.2
Eastern Sub-Saharan Africa	62.6	52.8	72.0	21.8	21.3	22.2	38.1	29.0	46.7	2.8	2.5	3.1	49.9	40.8	58.2	13.4	13.2	13.7	34.3	25.7	42.0	2.2	2.0	2.5
Central Sub-Saharan Africa	45.5	39.6	50.8	15.9	15.7	16.1	27.6	22.1	32.5	2.0	1.8	2.3	41.7	35.8	46.8	12.6	12.4	12.7	27.0	21.5	31.7	2.1	1.9	2.4
Western Sub-Saharan Africa	45.9	38.7	53.5	15.7	14.6	16.9	28.3	22.5	34.3	1.9	1.7	2.2	41.6	36.0	46.7	13.0	12.6	13.4	26.9	21.9	31.4	1.7	1.5	1.9

*T* total, *F* female, *M* male, *ICH* intracerebral hemorrhage, *IS* ischemic stroke, *SAH* subarachnoid hemorrhage

**Fig. 3:**
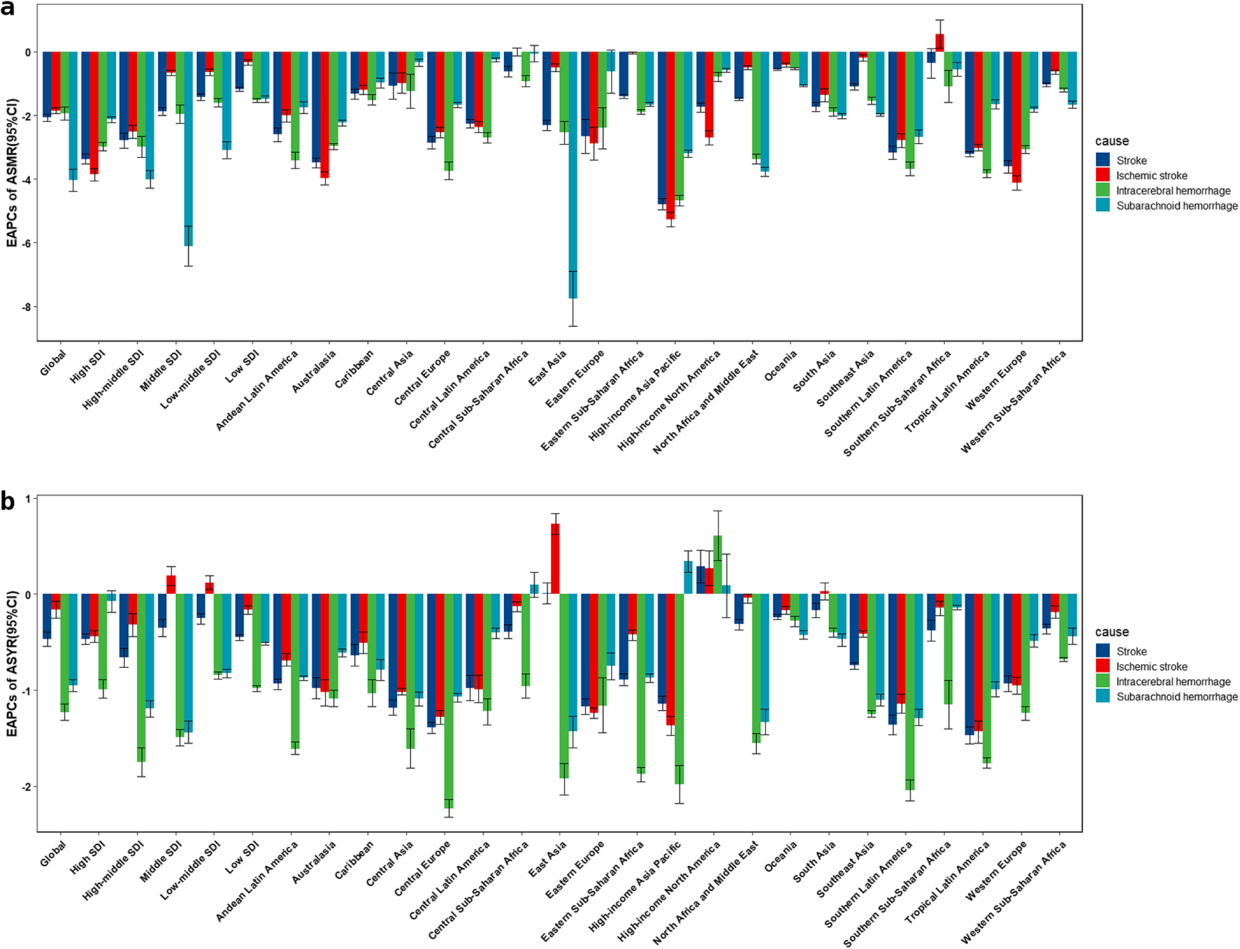
The EAPCs of ASMR and ASYR of diet-attributable stroke and subtypes from 1990 to 2019. *ASMR *age-standardized mortality rate, *ASYR *age-standardized YLDs rate, *YLDs *years lived with disability, *EAPC *estimated annual percentage change, *SDI *socio-demographic index

Figures 2b, d depicted each country or territory as a bubble, with the size representing the absolute burden and the Y coordinate displaying the EAPC from 1990 to 2019. A considerable negative correlation was observed between ASMR EAPCs and SDIs (*R* = -0.57, *p* < 0.001, Fig. 2b). Among the SDI quintiles, the high SDI quintile experienced the fastest reduction, with an EAPC of -3.37 (95% CI: -3.53, -3.21), while the low SDI quintile experienced the slowest decrease, with an EAPC of -1.17 (95% CI: -1.23, -1.11) (Fig. 3). Furthermore, ASYR EAPCs and SDIs were found to have a slightly negative correlation (*R* = -0.23, *p* = 0.001, Fig. 2d). Of note, the high-middle SDI quintile experienced the fastest decrease, with an EAPC of -0.66 (95% CI: -0.76, -0.56), while the low-middle SDI quintile had the slowest decrease, with an EAPC of -0.25 (95% CI: -0.31, -0.20) (Fig. 3).

### Stroke burden attributable to diet by GBD super regions

In 2019, Australasia, High-income North America, and Western Europe were the top three GBD 21 super regions with the lowest ASMRs, while Central Asia, Southeast Asia, and East Asia had the greatest ASMRs. Except for North Africa and Middle East, males had a higher burden of diet-related stroke death (Table 1). In terms of ASYR, the lowest rates were found in Andean Latin America, Central Latin America, and Caribbean, while the highest rates were found in East Asia, Southeast Asia, and Oceania. In all 21 super regions, females had a higher burden of diet-attributable stroke YLDs (Table 2).

The highest drop in ASMR was observed in High-income Asia Pacific, with an EAPC of -4.79 (95% CI: -4.97, -4.61), whereas the lowest decline was observed in Southern Sub-Saharan Africa, with an EAPC of -0.36 (95% CI: -0.82,0.10). Meanwhile, Tropical Latin America experienced the greatest decline in ASYR, with an EAPC of -1.47 (95% CI: -1.56, -1.38). However, two regions, high-income North America (EAPC of 0.29[95%CI:0.12,0.46]) and East Asia (EAPC of 0.01[95%CI: -0.1,0.12]), exhibited a slight rise during the study period (Fig. 3).

### Stroke burden attributable to diet by countries and territories

**Fig. 4:**
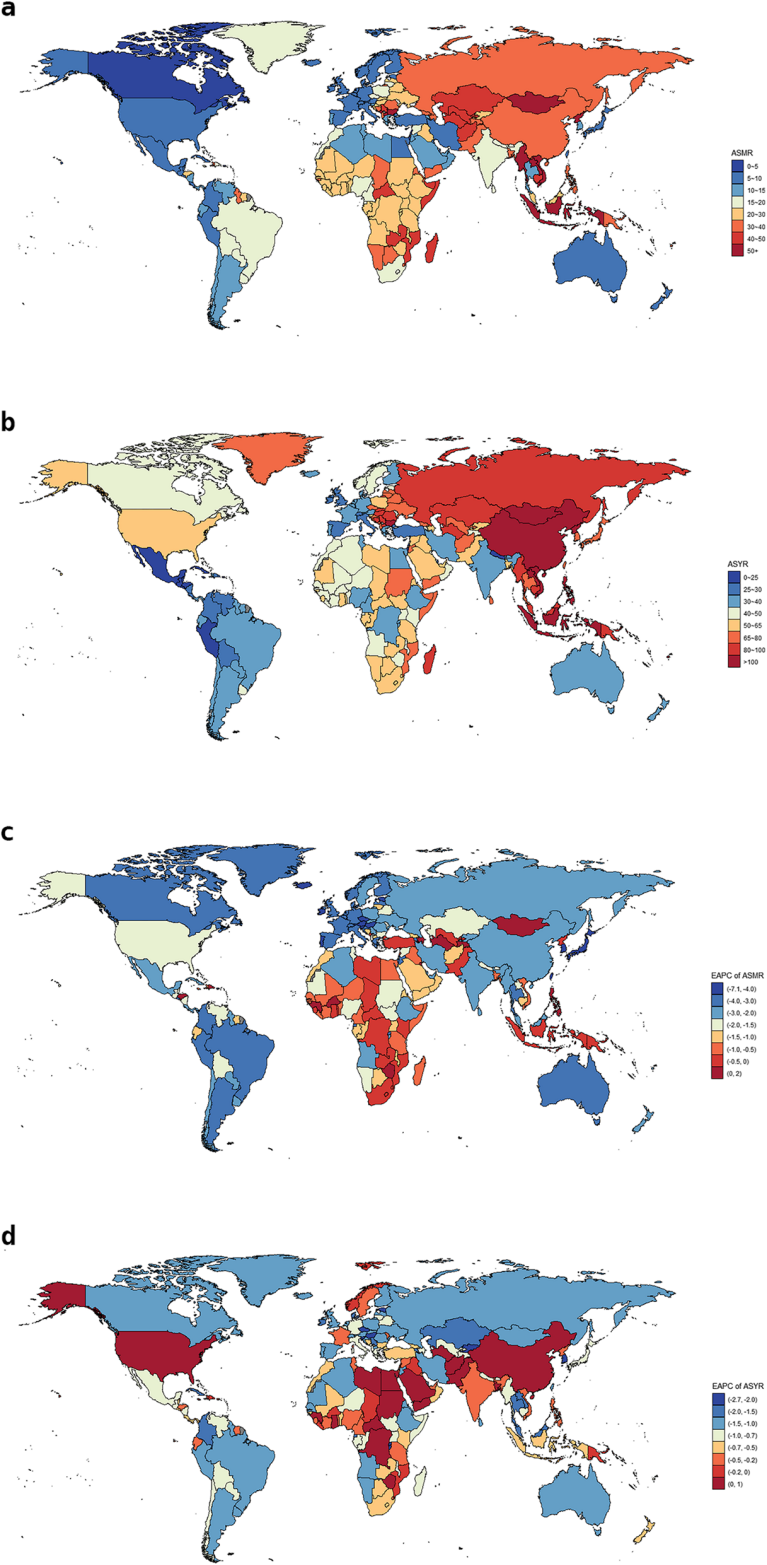
The global distribution of ASMR (**a**) and ASYR(**b**) of stroke attributable to diet for both genders in 204 countries and territories in 2019, and the corresponding EAPCs of ASMR(**c**) and ASYR(**d**) from 1990 to 2019. *ASMR *age-standardized mortality rate, *ASYR *age-standardized YLDs rate, *YLDs *years lived with disability, *EAPC *estimated annual percentage change

The ASR of stroke varied significantly across the world, as shown in the global heat map, with the highest ASMR observed in Mongolia with a value of 90.27 (95%UI: 64.65,120.51), followed by Solomon Islands and North Macedonia, all of which had an ASMR of more than 70.00 per 100,000 populations in 2019. Israel had the lowest ASMR, with a value of 3.93 (95%UI:2.51,5.99) (Fig. 4a). Vanuatu had the greatest ASYR of diet-attributable stroke, with a value of 132.56 (95% UI:83.40,193.79), followed by Indonesia and Kiribati, and Belize had the lowest ASYR, with a value of 18.27 (95% UI:11.06,26.88) (Fig. 4b).

At the national level, Estonia had the most dramatic decline in ASMR, with an EAPC of -7.09 (95CI: -7.69, -6.48), followed by South Korea and Singapore. Nonetheless, the Philippines had the strongest rising trend in ASMR, with an EAPC of 1.6 (95% CI:0.98,2.23), followed by Lesotho and Zimbabwe (Fig. 4c). Mauritius had the highest decline in ASYR, with an EAPC of -2.66 (95% CI: -2.85, -2.47), followed by Rwanda and Singapore. Meanwhile, Lebanon had the highest EAPC of ASYR, with an EAPC of 0.87 (95% CI:0.74,1.00), followed by Saudi Arabia and Guinea. We also found that certain populous countries, such as the United States, Bangladesh, Pakistan, and China, displayed an upward trend with EAPCs > 0(Fig. 4d).

### The burden and temporal trends of stroke subtypes attributable to diet

In 2019, regions with high SDI showed the lowest ASMRs for all stroke subtypes,while the highest ASMRs for ICH and SAH were observed in the low-middle SDI regions, and ISpeaked inhigh-middle SDI region (Table 1). ASYRs showed the most significant burden in middle SDI regions for IS and ICH, with the lowest in low SDI regions for IS and in high SDI regions for ICH. SAH's ASYR increased with SDI (Table 2).

Significant geographical disparities existed, with Eastern Europe recording the highest ASMR for IS, and Oceania for ICH and SAH. (Table 1). In terms of ASYR, East Asia had the highest rate for IS, Oceania for ICH, and High-income Asia Pacific for SAH. (Table 2).

A general downward trend in ASMR and ASYR was observed worldwide and across most SDI regions. Notably, the ASMR for IS increased in Southern Sub-Saharan Africa with an EAPC of 0.56 (95% CI: 0.12, 1.01), but remained stable in Central Sub-Saharan Africa, with an EAPC of 0 (95% CI: -0.12, 0.12) (Fig. 3). The highest EAPCs of ASYR were reported in East Asia for IS (0.73 [95% CI: 0.62,0.84]), High-income North America for ICH (0.61 [95% CI: 0.35,0.87]), and High-income Asia Pacific for SAH (0.34 [95% CI: 0.23,0.45]). Also, in High-income North America, the ASYRs for three subtypes showed an increasing trend (Fig. 3).

### Cross-country inequalities

**Fig. 5:**
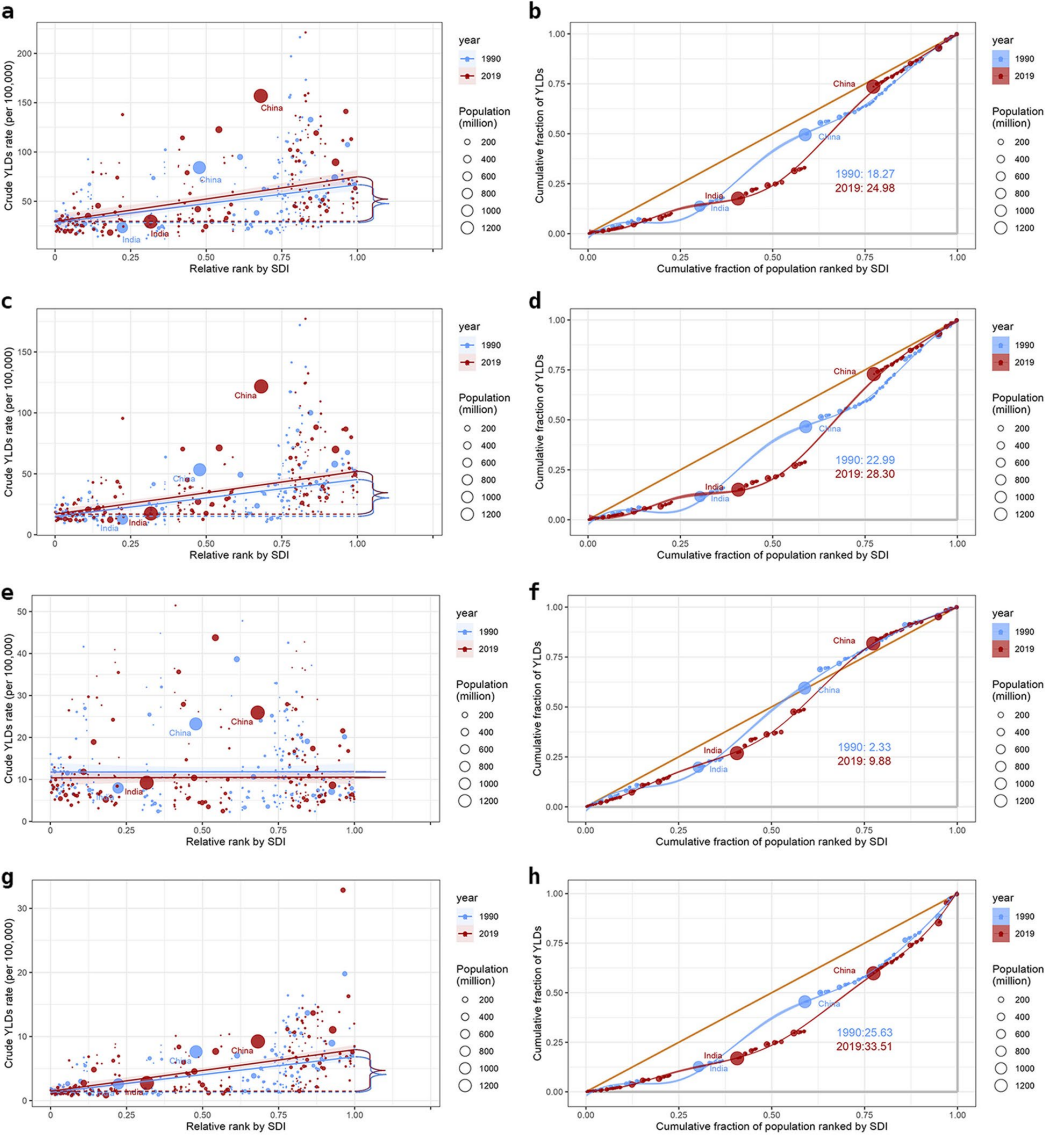
Health inequality regression curves and concentration curves for the YLDs of stroke (**a** &**b**), IS (**c** & **d**), ICH (**e** & **f**), and SAH (**g** & **h**) worldwide, 1990 and 2019. *YLDs *years lived with disability, *IS *ischemic stroke, *ICH *intracerebral hemorrhage, *SAH *subarachnoid hemorrhage

Significant absolute and relative SDI-related inequalities in the burden of stroke and subtypes were observed, with a disproportionate higher burden shouldered by countries with higher SDI. As illustrated by the SII, the gap in YLDs rate of stroke between the highest and the lowest SDI country increased from 38.35 (95% CI: 27.00, 49.71) in 1990 to 45.18 (95% CI: 32.72, 57.63) in 2019. Moreover, the RCI showed 18.27 (95% CI:15.82, 20.72) in 1990 and 24.98 (95% CI:20.78, 29.18) in 2019.The SII of IS increased from 30.22 (95% CI: 23.64, 36.80) in 1990 to 35.11 (95% CI: 27.66, 42.56) in 2019. The RCI of IS increased from 22.99 (95% CI:19.83, 26.14) in 1990 to 28.30 (95% CI:22.86, 33.74) in 2019. The SII of ICH increased from 0.11 (95% CI: -3.32, 3.54) in 1990 to 0.15 (95% CI: -2.73, 3.03) in 2019. The RCI of ICH increased from 2.33 (95% CI:1.94, 2.72) in 1990 to 9.88 (95% CI:8.37, 11.39) in 2019. The SII of SAH increased from 5.54 (95% CI: 4.34, 6.74) in 1990 to 6.48 (95% CI:5.12, 7.85) in 2019. The RCI of SAH increased from 25.63 (95% CI:21.97, 29.3) in 1990 to 33.51 (95% CI:28.62, 38.39) in 2019 (Fig. 5 & Table 3).

**Table 3:** Summary measures for SDI-related inequalities in YLDs of stroke and subtypes

Diseases	Health inequality metrics	Year	Value	95% CI
Stroke	SII	1990	38.35	27.00 to 49.71
		2019	45.18	32.72 to 57.63
	RCI	1990	18.27	15.82 to 20.72
		2019	24.98	20.78 to 29.18
IS	SII	1990	30.22	23.64 to 36.80
		2019	35.11	27.66 to 42.56
	RCI	1990	22.99	19.83 to 26.14
		2019	28.30	22.86 to 33.74
ICH	SII	1990	0.11	-3.32 to 3.54
		2019	0.15	-2.73 to 3.03
	RCI	1990	2.33	1.94 to 2.72
		2019	9.88	8.37 to 11.39
SAH	SII	1990	5.54	4.34 to 6.74
		2019	6.48	5.12 to 7.85
	RCI	1990	25.63	21.97 to 29.3
		2019	33.51	28.62 to 38.39

*SII* Slope index of inequality, *RCI* Relative Concentration index, *IS* ischemic stroke, *ICH* intracerebral hemorrhage, *SAH* subarachnoid hemorrhage, *CI* confidence interval

### Diet risks and their changes for YLDs of stroke and its subtypes

**Fig. 6:**
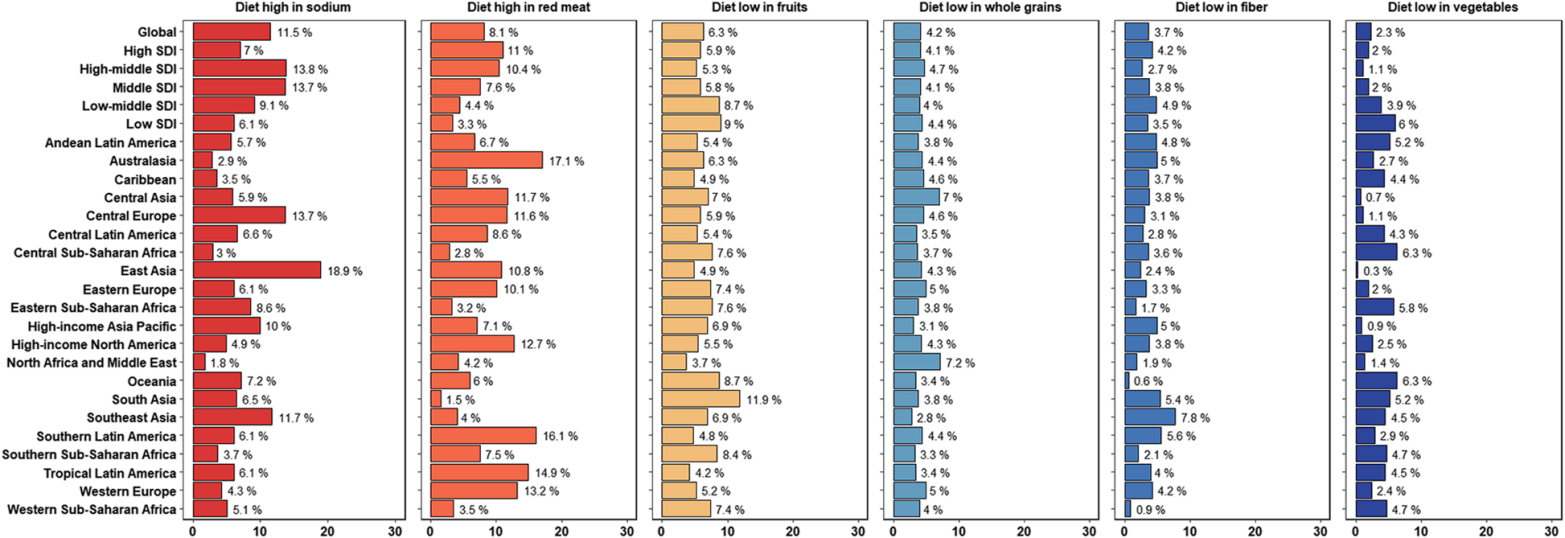
Proportion of stroke YLDs attributable to specific dietary components, for global,5 SDI and 21 GBD regions, 2019. *YLDs *years lived with disability, *SDI *socio-demographic index

In 2019, dietary risks significantly contributed to global stroke YLDs, with the highest impact seen from diets high in sodium (11.5%) and red meat (8.1%). Low fruit intake (6.3%), low whole grain intake (4.2%), low fiber intake (3.7%), and low vegetable intake (2.3%) were also notable risk factors (Fig. 6).

SDI regions showed distinct dietary risk profiles: high SDI regions were most affected by red meat, while high-sodium diets were a major concern in middle SDI regions. In low SDI regions, insufficient fruit and vegetable consumption, alongside high sodium intake, posed significant risks (Fig. 6).

The impact of dietary risks on stroke YLDs varied by region. High red meat consumption was a major risk in Australasia, Latin America, Western and Eastern Europe, North America, Central Asia, and the Caribbean. In contrast, East Asia, Southeast Asia, Central Europe, and the High-income Asia Pacific faced greater risks from high sodium intake. Low fruit intake predominantly affected South Asia, Oceania, and Sub-Saharan Africa. Additionally, the Middle East and North Africa were more burdened by insufficient whole grain consumption. Notably, East Asia, Australasia, and South Asia had the highest proportion of stroke YLDs attributable to high-sodium diets, high red meat diets, and low fruit diets, respectively (Fig. 6).

**Fig. 7:**
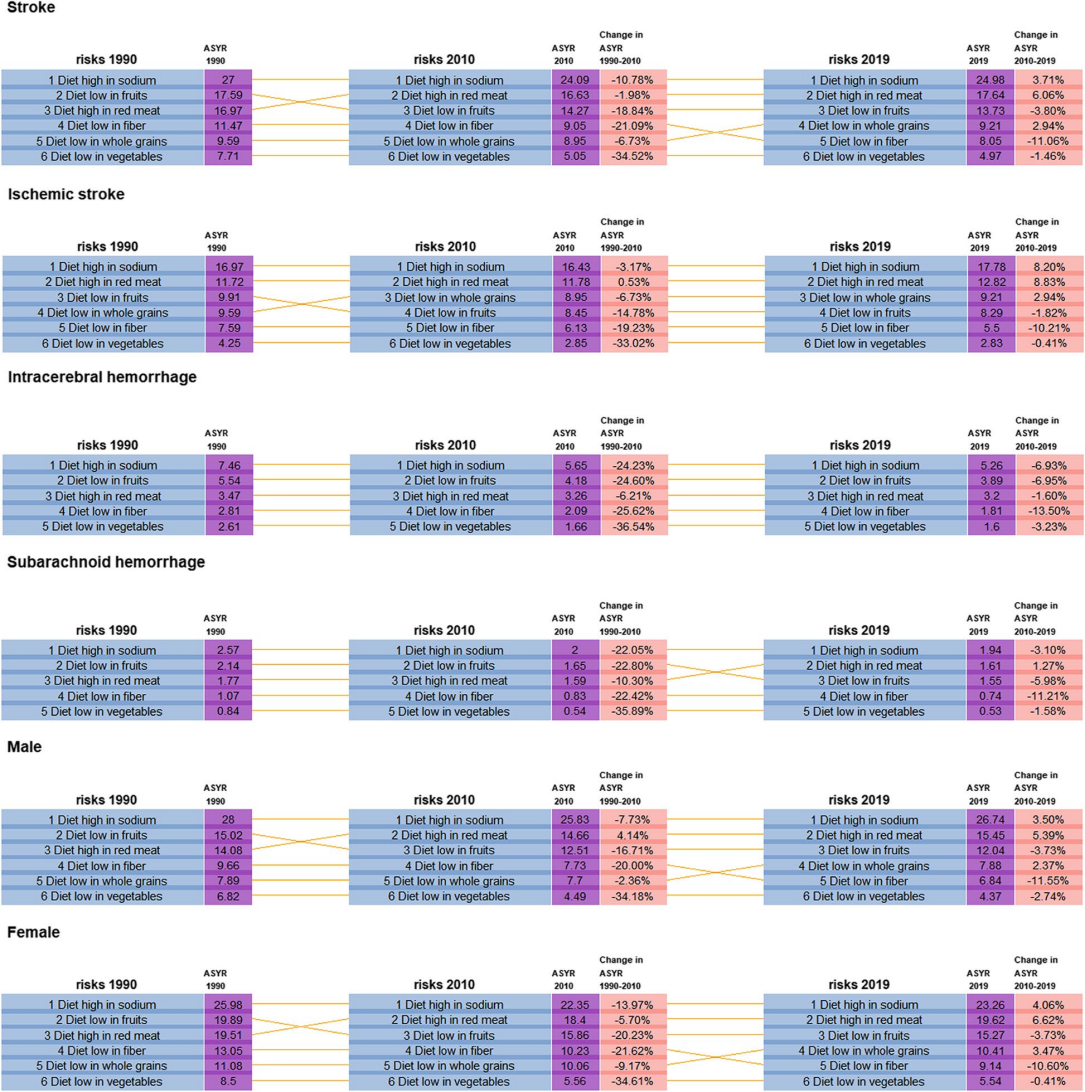
Leading dietary risks of YLDs of stroke in 1990, 2010 and 2019 with percentage change of ASYR by subtypes and genders. *ASYR *Age-standardized YLDs rate, *YLDs *years lived with disability

Over the decades, the ranking of dietary risks for stroke YLDs showed relative stability, with high red meat diets consistently ranking second and low whole grain diets rising in concern (Fig. 7). Diets low in fruits and fiber, on the other hand, fell in the standings. Despite an overall decrease in ASYRs due to dietary risks from 1990 to 2010, there was an upswing in ASYRs related to high sodium, red meat, and low whole grain diets between 2010 and 2019.

 Gender differences were observed, with females generally experiencing higher ASYRs than males, except for those attributable to high sodium diets. While females showed greater declines in ASYRs from 1990 to 2010, males had larger decreases from 2010 to 2019 (Fig. 7).

Regarding stroke subtypes, high sodium diets remained the primary dietary risk for YLDs across the decades. Low whole grain intake, particularly associated with IS, rose in ranking over the years. High red meat diets showed a steady increase in their contribution to IS YLDs, while the impact of dietary risks on ICH YLDs decreased overall. In 1990 and 2010, SAH had the same dietary risk rankings as ICH. However, in 2019, diets high in red meat surpassed diets low in fruits for the second ranking, with the ASYR increasing by 1.27% from 2010 to 2019 (Fig. 7).

## Discussion

This study comprehensively demonstrated the global burden of stroke and subtypes attributable to diet from 1990 to 2019. Worldwide, the burden of stroke attributable to dietary risks, in terms of deaths and YLDs, continues to increase. Over the same period, ASMR has been decreasing. However, the ASYR of stroke fluctuated but typically showed a declining tendency, and in 2014, the ASYR plummeted to its lowest point before showing an upward trend in subsequent years, implying a further increase in YLDs. This was consistent with previous research [13]. The rise in ASYR of stroke is mainly due to IS since the ASYR of ICH and SAH has continuously dropped. Factors such as population expansion, accelerated aging, enhanced screening programs, and improved diagnostic technologies contribute to the increase in stroke cases caused by diet [22]. On the other hand, higher living standards, increased self-awareness, faster disease detection, and easier access to effective therapies are associated with lower rates of diet-attributable stroke [23].

Of all stroke subtypes, ICH had the highest ASMR while IS had the highest ASYR. However, the reduction in IS for either ASMR or ASYR was minimal. In any given year, the ASMRs caused by stroke subtypes were significantly higher in males than in females. However, ASYRs were higher in females, except for ICH. Therefore, while males may be at a higher risk of dying, females may have a better chance of surviving a stroke with disability. The influence of sex chromosomes on human sex variations in aging and longevity may play a role in the different disease outcomes [24]. Additionally, the protective effects of estrogen, differences in dietary risk factor distribution, differences in vascular hemodynamics, and other pathophysiological factors may contribute to these differences [25]. Studies have indicated that males may have fewer positive attitudes and weaker self-regulation regarding fruit and vegetable intake and health consciousness when compared to females [26, 27]. If this trend continues, the gender disparity in dietary risk is likely to widen in the future. As such, it is essential to consider this significant difference in national prevention programs.

Age was found to be a significant risk factor, as reflected by the increase in crude mortality rate with age. In all age groups, males had a higher mortality rate compared to females, with females surpassing males only in the 95 plus age group. Conversely, the YLDs rate peaked at a relatively young age, particularly in males. This observation may be attributed to the a considerably longer lifespan of females and the higher mortality rate among the elderly population.

The ASMR of stroke related to dietary risk was found to have a negative association with the SDI. It was highest in the middle SDI region and decreased gradually to its lowest in the high SDI region. However, there was no significant correlation between ASYR and SDI. The EAPC of ASRs showed an inverse relationship with SDI, indicating faster declines in higher SDI regions. This finding is consistent with the results of the PURE study, which observed that despite a higher burden of cardiovascular risk factors in high-income countries, the incidence and mortality rates of stroke are lower in these countries compared to middle- and low-income nations [28]. High-income countries may be more inclined to promote healthy dietary patterns, such as the DASH diet and the Mediterranean diet, both of which have been demonstrated to reduce the risk of cardiovascular and cerebrovascular diseases.

There was an evident imbalance between different super regions, where the ASMRs of stroke caused by dietary risks were observed to be highest in Central Asia, Southeast Asia, and East Asia and lowest in Australasia, High-income North America, and Western Europe. Central Asia bore a significant burden of diet-related strokes, along with hypertension, diabetes, tobacco and alcohol consumption, which were identified as the main risk factors for stroke [29]. The region suffered from inadequate preventive care and a lack of education on stroke prevention and treatment, which further compounded the burden of stroke [30]. On the other hand, ASYRs were found to be highest in East Asia, Southeast Asia, and Oceania, while being lowest in Andean Latin America, Central Latin America, and the Caribbean. The rate of decline was faster in high-income regions, while Sub-Saharan Africa and Oceania witnessed a slow decrease.

Moreover, the scatter diagram including 204 countries and territories highlighted a growing tendency in nations with a higher burden of stroke YLDs, including the United States, Bangladesh, Pakistan, and China. Several countries, such as Estonia, South Korea, Singapore, Mauritius, and Czechia, witnessed larger losses in ASMR and ASYR. However, some countries such as Lesotho, Zimbabwe, Guinea, and Turkmenistan maintained positive trends in both ASMR and ASYR from 1990 to 2019. Mongolia, located in Central Asia, was one of the most affected countries with significant ASMR and ASYR. It is noteworthy that the rates of stroke mortality and YLDs in Mongolia were significantly higher than those reported in Western countries, especially for individuals aged between 35–55 years. Studies have linked the high burden of stroke in Mongolia to the low consumption of fruits and vegetables, which was found to be significantly associated with ischemic stroke [31].

As YLDs outnumbered deaths, we opted to utilize YLD metrics for further analysis. While IS remained the predominant stroke YLD category in 2019, ICH and SAH showed varying proportions in different income groups. For instance, East Asia and Eastern Europe should focus on IS, Oceania should prioritize ICH, and High-Income Asia Pacific must take note of SAH. Furthermore, our findings revealed that the burden of IS is on the rise in Sub-Saharan Africa.

All of the magnitudes of SII and RCI in 2019 were higher than those in 1990 for stroke and subtypes, suggesting that the SDI-related inequalities in the burden of strokes across countries exacerbated over time. Countries with higher SDI have low mortality rates, which may lead to more stroke survivors with disabilities. These people consume considerable medical resources and health care costs. Therefore, in countries with higher SDI, attention should be paid to disease prevention, especially through dietary factors.

Our analysis revealed that diets high in sodium and red meat, and low in fruits, whole grains, fiber, and vegetables were the primary dietary risk factors associated with stroke YLDs. In lower SDI countries in East and Southeast Asia, high sodium intake emerged as the top dietary risk for stroke. In contrast, European and American countries with higher SDIs were most impacted by high red meat consumption. For lower SDI countries in South Asia and Oceania, low fruit intake was the primary dietary risk factor associated with stroke.

China has consistently ranked high in terms of sodium consumption, making it the top contributor to adverse health outcomes affecting its citizens. High sodium consumption significantly increases the risk of hypertension, which is associated with ICH. Therefore, decreasing salt intake is a critical step towards stroke prevention in China. A research study showed that implementing a 10% reduction in sodium consumption across different countries could potentially prevent up to 5.8 million stroke-related disabilities over ten years, with densely populated countries, such as China, benefiting significantly from this effort [32].

A comprehensive review, which analyzed 122 qualified meta-analyses, revealed a negative association between red meat consumption and stroke [33]. Besides high in cholesterol, red meat also contains carnitine, which the gut microbiome converts into trimethylamine. The liver then oxidizes this compound into trimethylamine n-oxide (TMAO), which has been found to cause atherosclerosis in animals and significantly increase the risk of stroke [11].

Numerous studies have shown that a diet rich in fruits and vegetables is significantly correlated with lower blood pressure and reduced stroke mortality [34, 35]. Fruits and vegetables are typically high in fiber, micronutrients (such as vitamin C, folate, magnesium, and potassium), carotenoids, and phenolic compounds, all of which can significantly lower stroke risk [36]. This effect may be attributed, in part, to the presence of magnesium and potassium, which are known to decrease blood pressure. Vitamin C, which is predominantly found in fruits and vegetables, is an antioxidant that helps prevent oxidation of low-density lipoprotein cholesterol, suppress smooth muscle proliferation, and reduce systemic inflammation [36]. Additionally, high levels of circulatory lycopene and flavonoid intake have been linked to a reduced risk of stroke through antioxidative, anti-inflammatory, and antithrombotic actions, as well as improved endothelial health [37]. A study using 2019 GBD data for China found that poor fruit intake was associated with a higher risk of stroke mortality, particularly among older adults and males [6].

Consuming a diet low in whole grains has been found to be associated with an increased risk of ischemic stroke [38]. Previous studies have shown that grains such as corn, oats, and wheat contribute only 1.1% of total calories in the average diet. However, the higher cost, longer cooking times, and less appealing appearance of whole grains may deter some consumers from incorporating them into their diets [39, 40]. As a country with a high burden of ischemic stroke, China could potentially reduce its stroke risk by encouraging its population to switch from polished rice to whole grain rice. Although this may be a challenging shift to make, it could have a significant impact on stroke prevention. Targeting specific dietary factors can also be cost-effective and even cost-saving. For every US $1 invested in stroke prevention, there is an estimated return on investment of $10.9, making prevention efforts a wise investment in terms of public health and economic benefits [41].

Despite the overall decline in ASMR and ASYR attributed to stroke, some bad trends have emerged, arousing concern. Firstly, a discernible deceleration in the global trajectory towards the reduction of ASYR attributed to stroke due to dietary factors was observed during the past decade (2010–2019), with a notably slower pace compared to the preceding decades (1990–2010), particularly concerning the subtypes of ischemic stroke. Furthermore, between 2015 and 2019, the ASYR of females worldwide experienced a sizeable increase. Using the ARIMA forecasting model, it is projected that if current patterns persist, there will be 74.39 stroke YLDs per 100,000 population among females by 2050.Secondly, there has been an increasing trend of stroke ASYR observed in some high-income countries. Specifically, the ASYRs of the three subtypes exhibited an ascending tendency in High-income North America. In the United States, a worrisome trend in recent years has been the decline in hypertension awareness among individuals whose blood pressure is under control [42]. Thirdly, the ASYR of SAH is highest in High-income Asia Pacific and has been progressively increasing since 1990. The reason is not clear, but several factors include aging population, increase in high blood pressure, smoking, and a lack of public health awareness could contribute to this trend. All of these may have increased inequality between countries, with a greater burden on high SDI countries.

In actuality, the quality of one’s dietary choices is influenced by a multitude of complex and interrelated factors, encompassing the economics, culture, education, accessibility, and quality of food [43]. To address these issues, various dietary interventions have been recommended over the last few decades, such as media campaigns, menu and food labeling, price strategies, procurement rules for schools, workplace wellness initiatives and fiscal interventions (e.g. soda taxes), demonstrate that determined intervention can be successful in improving public health outcomes [44].

However, we acknowledge several limitations to our study. Firstly, our findings may be subject to memory bias resulting from reliance upon 24-h dietary recalls and Food Frequency Questionnaires. Due to the varied definitions and quantification of dietary risks across different regions of the world and the diverse array of foods ingested, it may be challenging to categorize dietary risks into distinct food or nutrient groups. Consequently, there may be potential under- or over-estimations of disease burdens attributed to diet. Secondly, we recognize that the majority of the data used in our analysis was predominantly derived from North American and European populations, with limited representation from other regions, particularly low-SDI regions, which may introduce biases into our results. Thirdly, we cannot completely avoid the potential for misclassification of stroke deaths, as stroke is a complex disease that is often accompanied by comorbid illnesses. This may result in an overestimation of the disease burden attributed to diet.

To mitigate the inequalities in stroke burden attributed to diet, we propose the following recommendations. Firstly, policymakers should advocate for dietary habits that reduce sodium and red meat intake while increasing the consumption of fruits, whole grains, fiber, and vegetables. This can be achieved through public health education campaigns, nutritional labeling, and tax incentives for healthy food options. Secondly, tailored nutrition programs should be designed to address the specific needs of different genders and age groups, with particular attention to males and the elderly population. Thirdly, high SDI countries should aim to decrease red meat consumption and encourage alternative protein sources such as fish and plant-based proteins. Middle SDI countries should focus on curbing sodium intake, while low-income countries should enhance the availability of fresh fruits and vegetables and reduce the marketing of unhealthy food products. Finally, establish monitoring systems to track trends in dietary patterns and stroke burden, evaluate the effectiveness of intervention measures, and adjust strategies based on data-driven insights.

## Conclusion

In summary, the present study found that suboptimal diets are a major contributor to strokes and the diet-related burden can vary substantially across year, age, gender, location and SDI. Countries with higher SDIs exhibited a disproportionately greater burden of stroke and its subtypes in terms of YLDs, and these disparities were found to intensify over time. Given the growing aging population worldwide, it is critical to enforce improved dietary practices, with a special emphasis on mortality drop in lower SDI countries and incidence decline in higher SDI countries.

## Data Availability

Data are available in a public, open access repository as follows: https://www.ghdx.healthdata.org/gbd-results-tool .

## Abbreviations

YLDs: Years lived with Disability
ASYR: Age-standardized YLDs rate
ASMR: Age-standardized mortality rate
ASR: Age-standardized rate
EAPC: Estimated annual percentage change
GBD: Global Burden of Diseases
DALYs: Disability-adjusted life years
ICH: Intracerebral hemorrhage
IS: Ischemic stroke
SAH: Subarachnoid hemorrhage
SDI: Socio-demographic index
UI: Uncertainty interval
CI: Confidence interval
WHO: World Health Organization
SII: Slope index of inequality
RCI: Relative Concentration index
HEAT: Health Equity Assessment Toolkit
DASH: Dietary Approaches to Stop Hypertension
